# How equitable are psychological therapy services in South East London now? A comparison of referrals to a new psychological therapy service with participants in a psychiatric morbidity survey in the same London borough

**DOI:** 10.1007/s00127-014-0900-6

**Published:** 2014-06-14

**Authors:** J. S. L. Brown, H. Ferner, J. Wingrove, L. Aschan, S. L. Hatch, M. Hotopf

**Affiliations:** 1 Psychology Department (PO77), Institute of Psychiatry, Kings College London, De Crespigny Park, London, SE5 8AF UK; 2 South London and Maudsley NHS Foundation Trust, London, UK

**Keywords:** Black and ethnic minorities, Equity, Psychological therapy, GP, Self-referral

## Abstract

**Purpose:**

Psychological therapy services are sometimes characterised as being small and inequitable, with an over-representation of white middle class women. The ‘Improving Access to Psychological Therapies (IAPT)’ initiative is a programme in England that attempts to make evidence-based therapies accessible to more people more equitably. The aim of this study is to assess whether an IAPT service is delivering an equitable service a London borough. Patients using services at the Southwark IAPT service (*n* = 4,781) were compared with a sub-group of participants in the South East London Community Health study (SELCOH) with diagnosable mental health problems and who were also resident in Southwark (*n* = 196).

**Methods:**

We compared Southwark IAPT patients and SELCOH participants on equity criteria of age, gender, ethnicity, occupational status and benefits status. To investigate if referral pathways influenced equity, patients referred by their general practitioner (GP pathway) (*n* = 3,738) or who self-referred (self-referral pathway) (*n* = 482) were compared with SELCOH participants.

**Results:**

Southwark IAPT patients significantly differed from SELCOH participants on all our equity criteria and similar differences were found with GP pathway patients. However, self-referrals did not differ from the SELCOH group on age, gender, ethnicity and benefit status.

**Conclusions:**

When compared to a community sample with diagnosable mental disorders, health disparities were found with the overall Southwark IAPT service and with GP pathway patients. Although unemployed people did access IAPT, fewer disparities were found with the self-referral pathway patients, suggesting that the IAPT self-referral pathway may be important in reducing inequitable access to services.

## Introduction

High quality care should ideally be both effective and provide equitable access for the population based on need [1]. In mental health, access to services is poor worldwide, with only about 30–40 % of individuals with mental disorders consulting their general practitioners (GPs) [2–4]. Access has been traditionally through GPs in publicly funded systems but has been problematic for several structural and individual reasons. GPs often fail to recognise mental disorders in patients [5]; may lack resources to manage them effectively [6, 7]; and individuals may be reluctant to consult GPs about emotional problems [8, 9]. In addition, access may be influenced by patient characteristics such as socioeconomic status and ethnicity [10, 11] which is a pattern of particular concern because inequitable access to services may perpetuate existing patterns of disadvantage [12].

Patterns of access to primary care services do show some problems of inequity. Epidemiological data indicate that up to 50 % of all adult mental health problems start by the mid-teens and 75 % by the mid-20s [13]. However, younger adults [14, 15] are much less likely to access services than those of working age [2, 14, 16]. Older people over 65 are also much less likely to access services [14]. Some ethnic inequities have also been found. GPs have been shown to be less likely to detect common mental health problems in black people compared to white people [10]. In addition, South Asians are less likely to consult, even after controlling for severity [2].

The role of psychological therapies is becoming increasingly recognised. In the UK, the National Institute for Health and Clinical Excellence (NICE) has advocated the use of evidence-based psychological therapies as first-line treatments for the treatment for depression and anxiety [17] In addition, the lack of change in the prevalence in neurotic disorders between 1993 and 2000, despite a two- to threefold increase in the prescription of antidepressants has led to recommendations that other treatments, notably psychological and social interventions, are needed to reduce prevalence [18].

However, psychological therapy services have sometimes been characterised as small and inequitable with white middle class women over-represented among service users. Following recommendations by Layard [19] who has emphasised the economic implications of untreated mental disorders such as depression and anxiety, the Improving Access to Psychological Therapy (IAPT) programme was introduced by the government-funded National Health Service (NHS) to increase the capacity of psychological therapy services in an equitable way. Under this programme, extra resources were invested in England to increase the number of psychological therapy staff as well as ensure NICE compliant standards were achieved through high-intensity psychological (HI) and low-intensity psychological (LI) training. Outcomes of treatment as well as service utilisation by different socio-demographic groups were also carefully monitored to assess equity.

Prior to the national roll-out, an analysis of users of the IAPT services in the two IAPT demonstration sites in Newham and Doncaster during the first 13 months of service had showed some patterns of inequity [20]. Less than 4 % of service users were under 18 or over 65 years of age, with the majority (58 %) aged between 25 and 44 years. A comparison of ethnicity in the local population, (independent of need), showed that whilst 61 % of the local population were from black and minority ethnic (BME) groups (38 % Asian, 20 % Black) [21], only 49 % of IAPT patients in Newham came from BME groups (25 % Asian, 17 % Black), supporting the previous findings about psychology services not being equitable [22]. However, data from the Newham service comparing GP and self-referrals indicated that the introduction of a self-referral route improved access for BME groups, with the proportion of people from black ethnic groups only coming through this route increasing to 22.2 %, whilst the proportion of black ethnic groups being referred by GPs remained low at 15.9 %. This supports work by Brown and colleagues [23] showing that the proportion of black participants self-referring to a psychological intervention was comparable to the rate in the local population. Sixty percent of those accessing the IAPT service were found to be female. In the demonstration sites, employment data were not complete (54 % Newham and 27 % Doncaster) but indicated that 31.7 % were on benefits. As these sites demonstrated the feasibility of the service, the new IAPT services were rolled out nationally.

The question that now arises is whether IAPT services are equitable in practice. Comparisons of socio-demographic and economic variables between patients using a service and the general population are often only partially informative because differences identified may be due to several factors including differences in geographical area (e.g. local, regional or national), access to services (e.g. capacity of services) or whether mental health need is measured and controlled for. It has been recommended that local data are essential to accurately examine inequities in mental health [24]. Equity here is defined as equal (or not unfairly restricted) access to health care services.

Southwark is a deprived borough in London [25]. It is also ethnically diverse with a greater number of Black-Caribbean residents but fewer South Asian residents than other areas of London [21]. The South East London Community Health Study (SELCoH) study is a community survey which was conducted to improve understanding of the health needs of the Southwark as well as Lambeth community, and make recommendations about improved service provision [26]. The present study will be the first to directly compare socio-demographic and socioeconomic characteristics of patients who are using an IAPT psychological therapy service in Southwark with individuals identified with mental health needs from the same catchment area (Southwark) who are participants in a community psychiatric morbidity study.

## Methods

### Design

This study was a cross-sectional analysis comparing individuals entering the Southwark IAPT service between April 2009 and December 2010 with those participating in the SELCoH survey between April 2008 and December 2010. Although Southwark IAPT service started operating in October 2008, only data from April 2009 was included to avoid possible inaccuracies arising from problems during the initial setting up of the service. The analysis compared Southwark IAPT patients with SELCOH participants scoring above the clinical threshold on the Clinical Interview Schedule—revised version (CIS-R) [27] (to be known as SELCOH12 participants).

### Study Aims

1. To compare all Southwark IAPT service users with SELCOH12 group on selected equity criteria, specifically, age, gender, ethnicity, employment status and benefit status.
2. To examine if referral pathways affect equity by comparing Southwark IAPT self-referral and GP referrals with SELCOH12 group on the same equity criteria.

We define equity as access to health services that is not unfairly restricted. Our hypotheses are that, when compared to SELCOH12 participants with probable mental disorder, the Southwark IAPT service will be shown to be equitable if:

1. There are no differences on equity criteria between SELCOH12 participants and Southwark IAPT patients, or if there are differences, these differences do not indicate restricted access.
2. There are no differences on equity criteria between SELCOH12 participants coming through either the GP pathway or the self-referral pathway, or if there are differences, these differences do not indicate restricted access.

### Southwark IAPT service

The Southwark IAPT service is part of the national IAPT programme which aims to provide evidence-based psychological therapies. In line with national IAPT policy, information on client demographic variables such as gender, age, ethnic category and employment status was routinely collected. Data were collected through the IAPT Psychological Therapy Patient Management System (IAPTus), a computerised system designed specifically for use in most IAPT services. Clinical staff uses it routinely to record service user data. Data from IAPTus were extracted, exported, and cleaned before comparison analyses were carried out. While the Southwark IAPT service is designed to take adults over 18, it also accepts a few 16 and 17 year olds. Because the location of the GP surgery the patient is registered with determines which IAPT service is used, a small proportion (2.2 %) of patients were non-Southwark residents. All patients who were referred by their GP or self-referred were included in the study.

### The South East London community health study (SELCoH)

A total of 1,698 adults aged 16 and over from 1,075 randomly selected households living in Southwark (*n* = 852) and Lambeth (*n* = 846) completed the survey between October 2008 and December 2010. Trained interviewers using a computer-assisted interview schedule carried out face-to-face interviews. The data collection procedure and questionnaire items are more fully described in Hatch et al. [26]. Of 2,359 participants eligible, 71.9 % participated. The achieved SELCOH study sample was representative of the catchment area with regard to the 2011 census demographic and socioeconomic indicators, with the exception of the study sample being slightly younger and including more students among the economically inactive (42 vs. 33.3 %). Weightings were applied to the raw data to allow for clustering and non-response. Only participants living in Southwark were used in this study because comparable data were only available for Southwark IAPT.

### Measures

Socio-demographic and socioeconomic data which were obtained from the Southwark IAPTus and SELCoHstudy databases.

#### Socio-demographic characteristics

Age, gender and ethnicity data were collected in both datasets but some recoding was necessary to ensure compatibility.

*Age* IAPTus age data were recoded from date of birth to age in years. To allow for comparisons with the literature, the following age categories were used: 16–24; 25–34; 35–44; 45–54; 55–64; 65+.

*Ethnicity* The 17 different ethnicity categories in IAPTus were recoded into the 9 categories used by SELCoH; White; Black-Caribbean; Black-African; Black Other; Indian; Pakistani; Bangladeshi; Chinese or Other Ethnic group.

#### Socioeconomic characteristics

Employment status and benefits status data were collected.

*Employment* The nine work categories used for SELCoH participants were collapsed into the six work categories used by IAPTus: employed full-time, employed part-time, unemployed, full-time student, retired or full-time homemaker or carer. The temporary sick or disabled category in SELCoH was recoded as Missing.

*Benefits* The SELCoH questionnaire listed 11 benefit categories and IAPTus listed 3 benefit categories. These were collapsed into a binary yes/no variable indicating receipt of one or more benefits. The ‘not stated’ category in IAPTus was recoded as missing/not stated.

#### Common mental disorder

The CIS-R [27] is a structured interview assessing psychiatric symptom status during the past year, and was used to assess severity of mental disorder among SELCOH participants. A threshold of CIS-R scores of 12 or above is conventionally used to indicate the presence of common mental disorder and used to define the SELCOH12 group.

#### Referral pathways

In IAPTus, sources of referral were categorised into GP referrals (GP pathway) or self-referrals (self-referral pathway) or other. Only the first two categories were used for the secondary analysis as the ‘Other’ category covered patients coming through a variety of pathways including referral from community mental health teams, specialist services and job centres.

### Analysis

Analyses were completed in Stata 11 [28]. Pearson’s *χ* ^2^ test was used for categorical variables, and the independent samples *t* test for the continuous age variable. All analyses of SELCoH data accounted for clustering by household which was inherent in the study design and weighted for non-response within households.

## Results

Table 1 gives details of the Southwark IAPT group (*n* = 4,781), the overall SELCOH group (*n* = 852) and the SELCOH12 group (*n* = 196). Of the SELCOH12 sample, 31.8 % had consulted their GPs in the previous year for a mental health problem. The most common diagnoses were depression (46.8 %), non-specific neurotic disorder (19.7 %) and generalised anxiety disorder (16.2 %). There was missing data, particularly for ethnicity (37 %), employment status (36.8 %) and benefit status (38 %) for GP referrals, largely because data on these variables were not available for those who did not come for their assessments. Clients whose data were missing were still included in the analysis.

**Table 1:** Descriptives and differences between SPTS and SELCOH12 group

	SELCOH	SELCOH 12 group	Southwark IAPT	Significance (IAPTv SELCOH 12)
*N*	852	196	4,781	

	Weighted mean	95 % CI	Weighted mean	95 % CI	Mean	95 % CI	
Age (years)							*t*(4,976) = 2.98, *p* = *0.003*
	44.1	(42.3–45.8)	42.1	(39.2–45.0)	37.7	(37.4–38.1)	

	Weighted %				95 % CI	Weighted %					95 % CI	%	95 %CI						*χ* ^2^ (*df*)
Age Categories (years)																			*χ* ^2^ (5) = 59.98, *p* < 0.001
16–24	19.4				(15.1–23.7)	21.0					(14.6–27.5)	15.3	(14.2–16.3)						
25–34	18.6				(15.8–21.5)	18.0					(12.6–23.3)	31.9	(30.6–33.2)						
35–44	17.3				(14.4–20.1)	17.6					(12.0–23.2)	25.2	(24.0–26.5)						
45–54	14.0				(11.6–16.4)	17.8					(12.3–23.3)	16.8	(15.7–17.8)						
55–64	13.1				(10.1–16.1)	14.2					(8.3–20.1)	6.6	(5.8–7.2)						
65+	17.6				(13.8–21.3)	11.4					(6.0–16.8)	4.3	(3.7–4.9)						
Gender (%)																			*χ* ^2^ (1) = 5.71, *p* = 0.0114
Male	33.8				(31.2–36.5)	27.6					(21.8–33.4)	35.7	(34.3–37.1)						
Female	66.2				(63.5–68.8)	72.4					(66.6–78.2)	64.3	(62.9–65.7)						
Ethnic Group (%)																			*χ* ^2^ (8) = 28.14, *p* = 0.0028
White	61.9				(57.4–66.4)	62.1					(54.0–70.3)	73.7	(72.1–75.2)						
Black-Caribbean	7.1				(5.0–9.3)	9.6					(4.7–14.6)	7.1	(6.1–8.0)						
Black-African	16.0				(12.6–19.3)	13.0					(7.2–18.7)	6.0	(5.1–6.9)						
Black Other	1.9				(0.7–2.7)	1.0					(0.0–2.5)	1.9	(1.4–2.4)						
Indian	1.2				(0.5–3.2)	2.2					(0.0–4.4)	0.9	(05–1.2)						
Pakistani	1.2				(0.1–2.3)	1.1					(0.0–2.1)	0.4	(0.1–0.6)						
Bangladeshi	0.5				(0.0–1.0)	0					0	0.5	(0.2–0.7)						
Chinese	1.3				(0.5–2.1)	0.9					(0–2.1)	1.0	(0.6–1.3)						
Other Ethnic group	8.4				(6.1–10.6)	10.1					(5.2–15.0)	8.7	(7.6–9.6)						
Employment Status (%)																			*χ* ^2^ (5) = 69.59, *p* < 0.001
Employed full-time	35.9				(32.1–39.7)	34.3					(26.5–42.1)	39.4	(37.6–41.0)						
Employed part-time	14.9				(12.4–17.5)	13.2					(7.9–18.6)	11.8	(10.6–12.9)						
Full-time student	15.9				(12.7–19.1)	14.4					(8.4–20.3)	6.5	(5.6–7.3)						
Unemployed	9.1				(7.0–11.2)	14.4					(8.7–20.2)	30.2	(28.5–31.8)						
Retired	19.8				(15.9–23.7)	19.3					(11.7–26.8)	6.2	(5.3–7.1)						
Full-time homemaker or carer	4.3				(2.8–5.7)	4.3					(1.2–7.5)	6.1	(5.2–6.9)						
Benefits Status (%)																			*χ* ^2^ (1) = 15.28, *p* < 0.001
Yes	24.9				(21.4–28.4)	41.4					(33.6–49.1)	28.5	(26.9–30.2)						
No	75.1				(71.6–78.6)	58.6					(50.9–66.4)	71.5	(69.8–73.1)						

### Comparison of Southwark IAPT patients (*n* = 4,781) and SELCOH12 group (*n* = 196)

Significant differences were found on all the equity criteria (Table 1).

*Socio-demographic characteristics* A significant difference in the mean age of the two groups was found, with Southwark IAPT patients being younger. When examining age categories, there were greater proportions of Southwark IAPT patients in the 25–34 and 35–44 age categories and fewer in the 55+ age groups.

Gender differences were also found. There was a greater proportion of male Southwark IAPT patients compared to SELCOH12 participants even though females formed the dominant group in both Southwark IAPT and the SELCOH12 groups.

Ethnic group differences were also significant. There was a greater proportion of the white ethnic group in the Southwark IAPT group and the proportion of Black-African participants was greater among SELCOH12 participants than among Southwark IAPT patients.

*Socioeconomic characteristics* Differences in employment status were found to be significant. Southwark IAPT patients were more likely to be unemployed and full-time homemakers or carers and less likely to be full-time students or retired people.

Finally, a smaller proportion of Southwark IAPT individuals were in receipt of benefits compared to SELCOH12 participants.

### Comparison of patients coming through the GP- and self-referral pathways with SELCOH12 group

#### GP-referral pathway (*n* = 3,738)

Descriptives and results of the analyses are shown in Table 2. Six differences between GP referrals and the SELCOH12 group were found and results were very similar to those found when all Southwark IAPT patients were compared to SELCOH12 participants. This may not be entirely surprising as GP referrals comprise 78.2 % of the Southwark IAPT referrals.

**Table 2:** Differences between SELCoH 12 group and GP and self-referral pathway groups

	SELCoH 12 group(1)	Southwark IAPT GP referrals (2)	Southwark IAPT self-referral(3)	Sig (1 v 2)	Sig (1v3)
*N*	196	3,738	482		

	Weighted Mean	95 % CI	Mean	95 % CI	Mean	95 % CI	*t*(*df*)	*t*(*df*)
Age (years)	42.1	(39.2–45.0)	37.1	(36.7–37.5)	40.8	(39.7–42.0)	*t*(3,932) = 3.41, *p* = 0.001	*t*(676) = 0.84, *p* = 0.401

	Weighted %	95 % CI	%	95 % CI	%	95 % CI	*χ* ^2^ (*df*)***	*χ* ^2^ (*df*)***
Age Category (years)							*χ* ^2^ = 65.47, *p* < 0.001	*χ* ^2^ (5) = 55.04, *p* < 0.001
16–24	21.0	(14.6–27.5)	16.5	(15.3–17.6)	7.3	(4.9–9.6)		
25–34	18.0	(12.6–23.3)	32.4	(30.9–33.9)	28.0	(24.0–32.0)		
35–44	17.6	(12.0–23.2)	25.5	(24.1–26.9)	27.4	(23.4–31.4)		
45–54	17.8	(12.3–23.3)	15.5	(14.4–16.7)	25.1	(21.2–29.0)		
55–64	14.2	(8.3–20.1)	6.1	(5.3–6.9)	7.9	(5.5–10.3)		
65+	11.4	(6.0–16.8)	4.0	(3.3–4.6)	4.4	(2.5–6.2)		
Gender (%)							*χ* ^2^ (1) = 6.25, *p* = *0.0081*	*χ* ^2^ (1) = 0.63, *p* = 0.405
Male	27.6	(21.8–33.4)	36.1	(34.6–37.7)	30.6	(26.5–34.8)		
Female	72.4	(66.6–78.2)	63.9	(62.3–65.4)	69.4	(65.2–73.5)		
Ethnic Group (%)							*χ* ^2^ (8) = 35.99, *p* < 0.001	*χ* ^2^ (8) = 4.75, *p* = 0.827
White	62.1	(54.0–70.3)	75.4	(73.7–77.1)	61.5	(56.4–66.6)		
Black-Caribbean	9.6	(4.7–14.6)	6.3	(5.3–7.3)	10.8	(7.5–14.0)		
Black-African	13.0	(7.2–18.7)	5.4	(4.5–6.4)	11.3	(8.0–14.7)		
Black Other	1.0	(0.0–2.5)	1.9	(1.3–2.4)	2.6	(0.9–4.2)		
Indian	2.2	(0.0–4.4)	0.7	(0.4–1.1)	1.4	(1.8–2.7)		
Pakistani	1.1	(0.0–2.1)	0.3	(0.01–0.5)	0.3	(0.0–0.8)		
Bangladeshi	0	0	0.5	(0.2–0.8)	0.3	(0.0–0.8)		
Chinese	0.9	(0–2.1)	1.1	(0.6–1.5)	0.6	(0.0–1.4)		
Other Ethnic Group	10.1	(5.2–15.0)	8.3	(7.2–9.4)	11.3	(8.0–14.7)		
Employment Status (%)							*χ* ^2^ (5) = 66.43, *p* < 0.001	*χ* ^2^ (5) = 59.54, *p* < 0.001
Employed full-time	34.3	(26.5–42.1)	41.9	(39.9–43.9)	32.1	(27.3–37.0)		
Employed part-time	13.2	(7.9–18.6)	12.2	(10.9–13.5)	10.0	(6.9–13.1)		
Full-time student	14.4	(8.4–20.3)	7.1	(6.0–8.1)	3.9	(1.9–5.9)		
Unemployed	14.4	(8.7–20.2)	27.3	(25.4–29.1)	40.4	(35.3–45.5)		
Retired	19.3	(11.7–26.8)	5.8	(4.9–6.7)	6.9	(4.3–9.6)		
Full-time homemaker or carer	4.3	(1.2–7.5)	5.8	(4.8–6.7)	6.7	(4.1–9.2)		
Benefits Status (%)							*χ* ^2^ (1) = 24.98, *p* < 0.001	*χ* ^2^ (1) = 0.92, *p* = 0.376
Yes	41.4	(33.6–49.1)	25.3	(23.5–27.1)	37.2	(32.1–42.3)		
No	58.6	(50.9–66.4)	74.7	(72.9–76.5)	62.8	(57.7–67.9)		

* Differences in numbers due to missing values

** SELCoH category of ‘temporary sick/disabled’ recoded as ‘missing’

*** Weighting accounted for

*Socio-demographic characteristics* GP referrals in the Southwark IAPT group were significantly younger than those in the SELCOH12 group, with greater proportions of Southwark IAPT patients in the 25–34 and 35–44 age categories and fewer in the older age groups.

Similarly, when compared with the SELCOH12 participants, there was a significantly greater proportion of male GP referrals relative to female. There were also significant ethnic differences with more Southwark IAPT patients who identified themselves as white and fewer Southwark IAPT patients who identified themselves as Black-African.

*Socioeconomic characteristics* Employment status differed between the two groups with more unemployed people, more full-time homemakers or carers, fewer students and fewer retired people in Southwark IAPT. GP referrals were also less likely to be in receipt of benefits than SELCOH12 participants.

#### Self-Referral Pathway (*n* = 482)

Self-referring patients and SELCOH12 participants were more similar and only two differences were found on the equity criteria.

*Socio-demographic characteristics* No significant difference between the mean age of the self-referrers and SELCOH12 groups were found (see Table 2). However, there was still a significant difference in age distribution between the two groups. There was a greater proportion of self-referrers in the 25–34 and 35–44 age groups.

No significant differences in gender distribution were found when comparing Southwark IAPT Self-Referrals with SELCOH12 participants, with a similar proportion of women coming through the self-referral route. Notably, no significant differences were found in ethnic group when self-referral and SELCOH12 groups were compared. A notable finding was that, compared to the SELCOH12 group, there were comparable proportions of individuals who identified themselves as Black-Caribbean or Black-African.

*Socioeconomic characteristics* Significant differences were found in employment status. There were significantly greater proportions of unemployed, and smaller proportions of students and retired people in the Self-Referrers group. However, proportions of individuals claiming benefits were similar between the two groups.

## Discussion

This study aimed to assess the extent of equity among patients accessing the Southwark IAPT Service, by comparing them with a representative group of individuals from the local population with diagnosable mental health problems. Results indicated that the overall Southwark IAPT group and GP referrals differed from community participants significantly on all six indices of equity examined. It needs to be noted that one of the differences found was in employment status, with a higher proportion of unemployed patients than SELCOH-12 participants. On the other hand, Southwark IAPT self-referrers did not differ significantly from the SELCOH 12 group on four indices (mean age, gender, and ethnicity and benefits status), but there were differences in two criteria, age (categories) and employment status. Again, more unemployed people were accessing the service through the self-referral route. This indicates that while both GP and self-referral pathways were accessible to unemployed people, the IAPT self-referral route may lead to a more equitable provision of psychological therapies compared to the GP-referral pathway, particularly with regard to ethnicity.

To our knowledge, this is the first time that the importance of the self-referral route in leading to greater equitable provision has been shown through a direct comparison of referrals to a psychological therapy service with a community sample with mental health problems from the same geographical area.

There may be several reasons why people came through the self-referral route rather than the more conventional GP-referral route. One possibility is that they felt able to self-refer to the Southwark IAPT Service as they did not have to face a potentially difficult consultation with the GP. Reasons cited in the literature about individuals’ difficulties include fearing the GP would not understand or have the time for mental health problems [8, 29]; differences in attitudes such as health beliefs such as perceptions of problems [30]; fearing being stigmatised [31]; or not wanting to be prescribed medication despite recognising its effectiveness [32]. Some of the above could apply to BME people who have often been reluctant to consult [2]. Another possible reason is that people coming through the self-referral route were more able to conceptualise their problems in a less medical way [9]. For example, those on benefits may have recognised their anxiety and depression as something the Southwark IAPT service could help with and self-referred. Another possible practical reason is that given a common obstacle to seeking help is that people often did not know about services [33], the publicity for the self-referral route that was placed in community settings, such as libraries, leisure centres and GP surgeries would have raised the profile of the IAPT therapy services among people who may previously have been reluctant to consult.

A key finding is that self-referrals did not significantly differ from the ethnic composition of SELCOH12 group participants while ethnic groups were under-represented in both the overall Southwark IAPT service and the GP-referral groups. This indicates that the self-referral route is more effective than the GP-referral pathway in ensuring equity in terms of ethnicity, particularly among Black-African people and Black-Caribbean people. It should be noted that ethnic differences between SELCOH12 and overall IAPT or GP referral patients were found for Black-African but not for Black-Caribbean participants, suggesting that Black-Caribbean participants are slightly more likely to consult their GPs than Black-Africans. These robust findings utilising a psychiatric morbidity survey support previous studies which have found BME self-referrals to be comparable to ethnicity rates in local populations where psychiatric morbidity has not necessarily been assessed [20, 23]. This finding therefore suggests that the self-referral route may be an important way of circumventing key barriers to access such as poorer GP detection of mental health problems of BME groups [10] and/or cultural differences in illness perceptions leading to reluctance to consult [9], or only being in touch with services at crisis points [34, 35].

The comparison of self-referrers with SELCOH12 participants suggests that more women with problems are coming through the self-referral pathway than through the GP pathway, indicating the self-referral pathway is allowing more equitable access. There is some evidence to suggest that male and female patients with common mental health problems are treated differently by GPs. For instance, Hyde and colleagues [36] found that males were more likely to be offered active treatment for depression by GPs than females. These differences may be due to a number of factors such as concern regarding higher suicide rates in young men or that they are considered less likely to return for follow-up appointments than women, and are therefore managed more actively at the initial consultation [37].

Our results indicated that adults in mid-life had more access to the service than young and older adults. Similar findings have been shown in comparisons with census data [16, 20]. The 16–24 category, and those aged 55 years and above therefore still remain under-represented.

More unemployed people are coming through into the Southwark IAPT service compared to the local population with mental health problems. Whilst it may be that there is greater need, our analysis where we controlled for need by only including those with CIS-R scores above the threshold, would indicate that this may not be the case. It may be that the service provides additional support for those people with mental health problems but who may lack a social support network. It may also be that unemployed people have more time to attend services or be more likely to be referred or self-refer.

In terms of receiving benefits, the proportion receiving benefits among self-referrals (37.2 %) was similar to that of the SELCOH12 group (41.4 %), and higher than those coming along the GP pathway (25.3 %). It is possible that this pattern may be the result of self-referrers being more likely to conceptualise their social problems as benefitting from psychological help, whereas those on benefits who consulted their GPs may have seen their problems as amenable to help from a doctor. It is also important to note that those who were working can still claim benefits, such as housing and child benefits.

### Limitations

There are several limitations. Whilst overall group sizes were large, group sizes for some ethnic categories were small so that conclusions about the accessibility of the service for some of these groups need to be cautious. Another limitation was that while it was necessary to provide accurate comparisons, the study only focussed on one urban London borough, Southwark, over a limited time period. Hence, the degree to which results may be generalisable to other IAPT services in more rural areas and other time frames needs further exploration. However, there is no reason to suspect that self-referral would reduce inequitable access in some areas and not in others. IAPT is a relatively new service, and it may be that as public knowledge about the service, and particularly self-referral increases, inequalities in access may decrease.

Differences in data collection may also account for some of the observed differences in findings. For example, the IAPT data are obtained from patients completing questionnaires on their own while the SELCoH data were collected by trained interviewers. In addition, some categories did not precisely overlap, especially in the benefits, age and employment categories. SELCOH included a temporary off-work/sick option while IAPTus did not. Another difference was that IAPTus asked whether people were full-time homemakers or carers, while the SELCoH equivalent referred to full-time homemakers only.

One limitation of this study is that there is no direct comparison of the psychiatric need of the Southwark IAPT service patients and of SELCOH participants. Nor do we have information about all the services received. For example, students who do not present to IAPT may use their own counselling services, or go to their GP where their parents’ home is. The IAPT sample will probably include a proportion of patients who would not meet threshold for CIS-R. We are also making the assumption that those who score about the clinical threshold on the CIS-R are suitable for IAPT. Another limitation is that the CIS-R has not been validated with culturally diverse populations, and so these results should be viewed with some caution. Finally as in any community survey SELCOH had the problem of incomplete participation. It is likely that those with mental disorders are under-represented, and it is probable that certain groups with mental disorder (e.g. some ethnic minorities) are particularly under-represented [38].

### Service implications

The IAPT service clearly provides a service for unemployed people coming through the GP or self-referral route. However, the results from this study also show that the use of self-referral in the IAPT allows for more equitable provision because there are fewer differences with the SELCOH-12 community group with regard to BME background, gender and people on benefit. This means that the self-referral route to IAPT may be a way of not adding to, and possibly reducing, the pattern of further disadvantage that inequity may otherwise contribute [12].

While some IAPT services were initially reluctant to use self-referral for fear of being overwhelmed by the number of people referring themselves, the relatively small number of people coming through, with 12 % in this study, and the results obtained justifies the decision for the system to be introduced and even expanded.

However, the self-referral route does not altogether solve the inequity problem as younger people (students and the younger age group (18–24)) and older people (retired and those over 45) still do not access the service, whether through GP referral or through self-referral. It will be important to explore further what the barriers to treatment may be, and specifically how far they are related to service delivery or individual preferences. Following from that, it may be possible to develop interventions to attract those groups. It will be important to address this issue given the importance of early intervention with young people [39] and the high costs of untreated mental health problems in older adults [40].

## Conclusion

The findings from this study would suggest that the IAPT service has been partially successful in providing an equitable service in Southwark. It clearly provides a service that unemployed people can access. Nevertheless, when compared to people in the local community with common mental health problems on other indices of equity, clear disparities were found with the overall Southwark IAPT service and GP referrals in particular. Even though relatively few people use this pathway into care at the moment, the self-referral pathway has been shown to help provide a more equitable provision in the IAPT service.
